# A Comparison of the Proteomic Expression in Pooled Saliva Specimens from Individuals Diagnosed with Ductal Carcinoma of the Breast with and without Lymph Node Involvement

**DOI:** 10.1155/2009/737619

**Published:** 2009-12-20

**Authors:** Charles F. Streckfus, Karen A. Storthz, Lenora Bigler, William P. Dubinsky

**Affiliations:** UTHSC—Dental Branch, CCTS—Salivary Proteomics Core, 6516 M.D. Anderson Blvd., Room 4.133f, Houston, TX 77030, USA

## Abstract

*Purpose*. The objective was to compare the salivary protein profiles of saliva specimens from individuals diagnosed with invasive ductal carcinoma of the breast (IDC) with and without lymph node involvement. *Methods*. Three pooled saliva specimens from women were analyzed. One pooled specimen was from healthy women; another was from women diagnosed with Stage IIa IDC and a specimen from women diagnosed with Stage IIb. The pooled samples were trypsinized and the peptide digests labeled with the appropriate iTRAQ reagent. Labeled peptides from each of the digests were combined and analyzed by reverse phase capillary chromatography on an LC-MS/MS mass spectrometer. *Results*. The results yielded approximately 174 differentially expressed proteins in the saliva specimens. There were 55 proteins that were common to both cancer stages in comparison to each other and healthy controls while there were 20 proteins unique to Stage IIa and 28 proteins that were unique to Stage IIb.

## 1. Introduction

Clinicopathologic factors such as histologic type, tumor size, tumor grade, HER-2/*neu *over-expression, hormone receptor status, and lymph node involvement are recognized as having prognostic use in breast cancer management [1–4]. Collectively assessed, axillary lymph node metastasis is the most important prognostic factor predicting breast cancer patient survival [5–7]. Currently, the best predictor of axillary lymph node metastasis is the presence or absence of metastasis in the sentinel lymph node. 

Current methodologies for this assessment are limited to axillary lymph node dissection and sentinel lymph node biopsy; however, these procedures are not without risks. For example, level I and level II axillary lymph node dissection can be associated with upper extremity lymph edema, wound complications, or nerve injury in a significant proportion of patients. Although sentinel lymph node biopsy is far less morbid than axillary lymph node dissection, it is not without risks or morbidity [8, 9]. Sentinel lymph node biopsy, despite its low but measurable false negative rate, provides no information about the presence of additional nonsentinel lymph node metastasis, which may occur in 40% to 70% of cases [10–12]. As a consequence, newer, more accurate, and less invasive means of predicting axillary lymph node metastasis would greatly improve breast cancer patient management and quality of life.

Morphological mimicry among human malignancies is a well-known histopathological phenomenon [11]. This is especially true in the case of the neoplastic ductal tissues of the breast and the salivary glands where immunostaining revealed the presence of Her2/*neu* (c-*erb*B-2), progesterone receptor, androgen receptor, and GCDFP-15 among these diseased tissues [11–14]. These studies suggest that similar molecular pathway dysfunctions may also be common within both tissues types [15].

Consequently, research has been performed concerning the presence of cancer related proteins and protein alterations in the secretory by products of these tissues, that is, saliva and nipple aspirate fluid (NAF) [16, 17]. Single analyte ELISA-based analyses yielded the presence of soluble Her2/*neu* protein in both saliva and NAF; Her2/*neu* concentrations were found to be elevated in both fluids secondary to the presence of carcinoma of the breast [16, 17]. Remarkably, Her2/*neu* protein concentrations were also elevated in the contralateral healthy breast within the same subject. Collectively, this line of research suggests that cancer-related cellular signaling may affect healthy exocrine tissues and result in alterations of their secretory by products. Likewise, EGFR and TNF-*α* were also found to be present in both fluids and altered in the presence of malignant breast disease [18]. Adding further support to this concept, both saliva and NAF were analyzed using mass spectrometry [19–21]. These fluid analyses yielded striking similarities with respect to their protein profile in health and were altered in the presence of neoplastic disease [19, 21].

Capitalizing on the potential of this possible relationship, significant salivary protein profile comparisons and alterations were reported in early stage breast cancer [21]. As a consequence, the purpose of this paper is to report saliva alterations secondary to late stage IDC with a focus concerning lymph node and nonlymph node involvement among the IDC cohorts.

## 2. Methods

### 2.1. Design

The investigators protein profiled three pooled, stimulated whole saliva specimens. One specimen consisted of pooled saliva from 10 healthy subjects, another specimen was a pooled saliva specimen from 10 Stage IIa (T_2_N_0_M_0_) invasive ductal carcinoma patients (IDC), and the third pooled specimen was from 10 subjects diagnosed with Stage IIb (T_2_N_1_M_0_) invasive ductal carcinoma [22]. The cancer cohorts were estrogen, progesterone, and Her2/neu receptor status negative as determined by the pathology report. Histological grade was not available for this study. The subjects were matched for age and race and were nontobacco users. 

The participating subjects were given an explanation about their participation rights and signed an IRB consent form. The saliva specimens and related patient data are nonlinked and bar coded in order to protect patient confidentiality. This study was performed under the UTHSC IRB approved protocol number HSC-DB-05-0394. All procedures were in accordance with the ethical standards of the UTHSC IRB and with the Helsinki Declaration of 1975, as revised in 1983.

### 2.2. Saliva Collection and Sample Preparation

Stimulated whole salivary gland secretion is based on the reflex response occurring during the mastication of a bolus of food. Usually, a standardized bolus (1 gram) of paraffin or a gum base (generously provided by the Wrigley Co., Peoria, IL) is given to the subject to chew at a regular rate. The individual, upon sufficient accumulation of saliva in the oral cavity, expectorates periodically into a preweighed disposable plastic cup. This procedure is continued for a period of five minutes. The volume and flow rate is then recorded along with a brief description of the specimen's physical appearance [23]. The cup with the saliva specimen is reweighed and the flow rate determined gravimetrically. The authors recommend this salivary collection method with the following modifications for consistent protein analyses [24]. A protease inhibitor from Sigma Co (St. Louis, MI, USA) is added along with enough orthovanadate from a 100 mM stock solution to bring its concentration to 1 mM. The treated samples were centrifuged for 10 minutes at top speed in a table top centrifuge. The supernatant was divided into 1 mL aliquots and frozen at −80°C. 

### 2.3. LC-MS/MS Mass Spectroscopy with Isotopic Labeling

Recent advances in mass spectrometry, liquid chromatography, analytical software, and bioinformatics have enabled the researchers to analyze complex peptide mixtures with the ability to detect proteins differing in abundance by over 8 orders of magnitude [25]. One current method is isotopic labeling coupled with liquid chromatography tandem mass spectrometry (IL-LC-MS/MS) to characterize the salivary proteome [26]. The main approach for discovery is a mass spectroscopy-based method that uses isotope coding of complex protein mixtures such as tissue extracts, blood, urine, or saliva to identify differentially expressed proteins [27]. The approach readily identifies changes in the level of expression, thus permitting the analysis of putative regulatory pathways providing information regarding the pathological disturbances in addition to potential biomarkers of disease. The analysis was performed on a tandem QqTOF QStar XL mass spectrometer (Applied Biosystems, Foster City, CA, USA) equipped with an LC Packings (Sunny vale, CA, USA) HPLC for capillary chromatography. The HPLC is coupled to the mass spectrometer by a nanospray ESI head (Protana, Odense, Denmark) for maximal sensitivity [16]. The advantage of tandem mass spectrometry combined with LC is enhanced sensitivity and the peptide separations afforded by chromatography. Thus even in complex protein mixtures MS/MS data can be used to sequence and identify peptides by sequence analysis with a high degree of confidence [21, 25, 26, 28]. 

Isotopic labeling of protein mixtures has proven to be a useful technique for the analysis of relative expression levels of proteins in complex protein mixtures such as plasma, saliva urine, or cell extracts. There are numerous methods that are based on isotopically labeled protein modifying reagents to label or tag proteins to determine relative or absolute concentrations in complex mixtures. The higher resolution offered by the tandem Qq-TOF mass spectrometer is ideally suited to isotopically labeled applications [21, 26, 29, 30]. 

Applied Biosystems recently introduced iTRAQ reagents [26, 29, 30], which are amino reactive compounds that are used to label peptides in a total protein digest of a fluid such as saliva. The real advantage is that the tag remains intact through TOF-MS analysis; however, it is revealed during collision-induced dissociation by MSMS analysis. Thus in the MSMS spectrum for each peptide there is a fingerprint indicating the amount of that peptide from each of the different protein pools. Since virtually all of the peptides in a mixture are labeled by the reaction, numerous proteins in complex mixtures are identified and can be compared for their relative concentrations in each mixture. Thus even in complex mixtures there is a high degree of confidence in the identification.

### 2.4. Salivary Protein Analyses with iTRAQ

Briefly, the saliva samples were thawed and immediately centrifuged to remove insoluble materials. The supernatant was assayed for protein using the Bio-Rad protein assay (Hercules, CA, USA) and an aliquot containing 100 *μ*g of each specimen was precipitated with 6 volumes of −20°C acetone. The precipitate was resuspended and treated according to the manufacturers instructions. Protein digestion and reaction with iTRAQ labels was carried out as previously described and according to the manufacturer's instructions (Applied Biosystems, Foster City, CA). Briefly, the acetone precipitable protein was centrifuged in a table—top centrifuge at 15,000 × g for 20 minutes. The acetone supernatant was removed and the pellet resuspended in 20 uL dissolution buffer. The soluble fraction was denatured and disulfides reduced by incubation in the presence of 0.1% SDS and 5 mM TCEP (tris-(2-carboxyethyl) phosphine)) at 60°C for one hour. Cysteine residues were blocked by incubation at room temperature for 10 minutes with MMTS (methyl methane-thiosulfonate). Trypsin was added to the mixture to a protein : trypsin ratio of 10 : 1. The mixture was incubated overnight at 37°C. The protein digests were labeled by mixing with the appropriate iTRAQ reagent and incubating at room temperature for one hour. On completion of the labeling reaction, the four separate iTRAQ reaction mixtures were combined. Since there are a number of components that can interfere with the LCMSMS analysis, the labeled peptides are partially purified by a combination of strong cation exchange followed by reverse phase chromatography on preparative columns. The combined peptide mixture is diluted 10-fold with loading buffer (10 mM KH_2_PO_4_ in 25% acetonitrile at pH 3.0) and applied by syringe to an ICAT Cartridge-Cation Exchange column (Applied Biosystems, Foster City, CA) column that has been equilibrated with the same buffer. The column is washed with 1 mL loading buffer to remove contaminants. To improve the resolution of peptides during LCMSMS analysis, the peptide mixture is partially purified by elution from the cation exchange column in 3 fractions. Stepwise elution from the column is achieved with sequential 0.5 mL aliquots of 10 mM KH2PO4 at pH 3.0 in 25% acetonitrile containing 116 mM, 233 mM, and 350 mM KCl, respectively. The fractions are evaporated by Speed Vac to about 30% of their volume to remove the acetonitrile and then slowly applied to an Opti-Lynx Trap C18 100 uL reverse phase column (Alltech, Deerfield, IL) with a syringe. The column was washed with 1 mL of 2% acetonitrile in 0.1% formic acid and eluted in one fraction with 0.3 mL of 30% acetonitrile in 0.1% formic acid. The fractions were dried by lyophilization and resuspended in 10 uL 0.1% formic acid in 20% acetonitrile. Each of the three fractions was analyzed by reverse phase LCMSMS. 

### 2.5. Reverse Phase LCMSMS

The desalted and concentrated peptide mixtures were quantified and identified by nano-LCMS/MS on an API QSTAR XL mass spectrometer (ABS Sciex Instruments) operating in positive ion mode. The chromatographic system consists of an UltiMate nano-HPLC and FAMOS autosampler (Dionex LC Packings). Peptides were loaded on a 75 cm x 10 cm, 3 mm fused silica C18 capillary column, followed by mobile phase elution: buffer (A) 0.1% formic acid in 2% acetonitrile/98% Milli-Q water and buffer (B): 0.1% formic acid in 98% acetonitrile/2% Milli-Q water. The peptides were eluted from 2% buffer B to 30% buffer B over 180 minutes at a flow rate 220 nL/min. The LC eluent was directed to a NanoES source for ESI/MS/MS analysis. Using information-dependent acquisition, peptides were selected for collision induced dissociation (CID) by alternating between an MS (1 second) survey scan and MS/MS (3 seconds) scans. The mass spectrometer automatically chooses the top two ions for fragmentation with a 60-second dynamic exclusion time. The IDA collision energy parameters were optimized based upon the charge state and mass value of the precursor ions. In each saliva sample set there are three separate LCMSMS analyses.

### 2.6. Bioinformatics

The accumulated MSMS spectra are analyzed by ProQuant and ProGroup software packages (Applied Biosystems) using the SwissProt database for protein identification. The ProQuant analysis was carried out with a 75% confidence cutoff with a mass deviation of 0.15 Da for the precursor and 0.1 Da for the fragment ions. The ProGroup reports were generated with a 95% confidence level for protein identification. Protein Pilot software package was used to assess the data produced from the mass spectrometry analyses. The Venn diagrams were constructed using the NIH software program (http://ncrr.pnl.gov). Graphic comparisons with log conversions and error bars for protein expression were produced using the ProQuant software. Descriptive statistics were performed using SPSS statistical software.

## 3. Results

Tables 1–5 summarize the results of the mass spectrometry analysis of the pooled salivary specimens and illustrate protein comparisons between Stage IIa versus healthy and Stage IIb versus healthy. The results identified and compared approximately 174 differentially expressed proteins in the saliva specimens. Of the 174 proteins, 158 (91%) were significant at an alpha level of *P *< .05 with a 95% confidence level. The mean percent peptide coverage for the complete panel proteins was 63.5% (±19.6) with a range of 35% to 96.5% coverage. The median value was 67.7% coverage.

**Figure 1:**
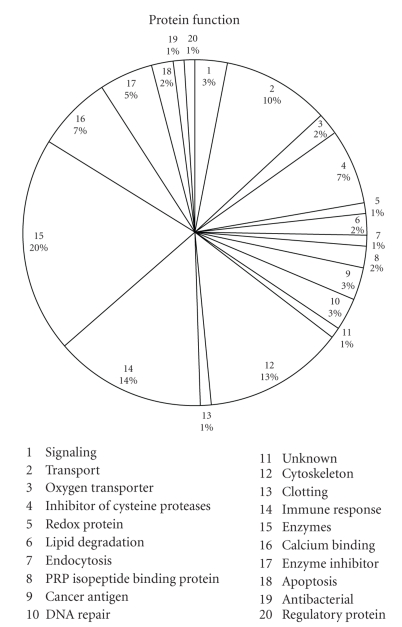
It represents protein function.

The pie chart in Figure 1 illustrates the percentage of proteins according to protein function. There were 55 proteins that were common to both cancer stages in comparison to each other while there were 20 proteins unique to Stage IIa and 28 proteins that were unique to Stage IIb (Table 1). 

**Figure 2:**
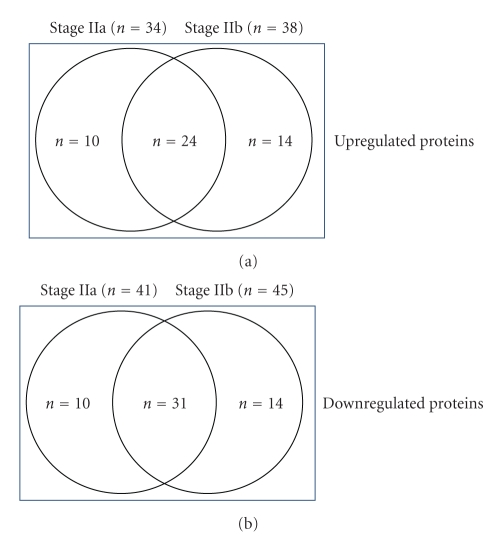
It represents a Venn diagram of the overlapping proteins within stage IIa and stage IIb.

**Figure 3:**
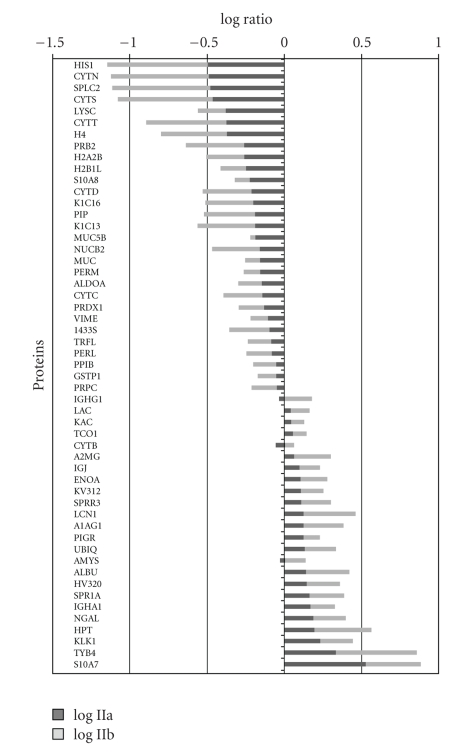
Differential expressions of salivary proteins common to both stage IIa and stage IIb.

**Figure 4:**
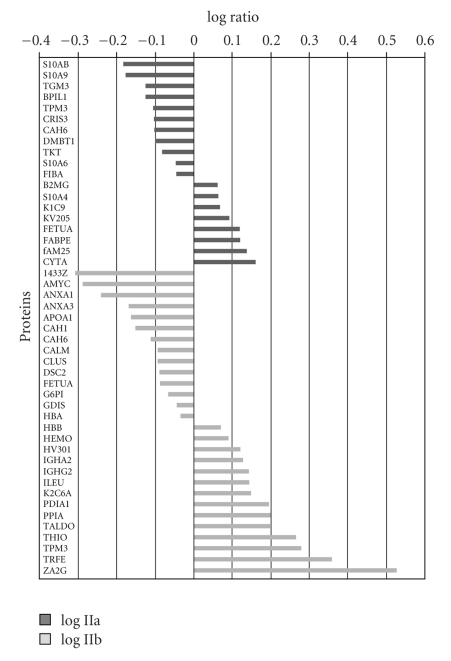
Differential expressions of salivary proteins that were not common to both stage IIa and stage IIb.

Figure 2 represents a Venn diagram of the overlapping proteins between the three groups of women. Figure 3 illustrates the comparison of the log ratio of the relative intensity (cancer/control) of the proteins which were common to both Stage IIa and Stage IIb while Figure 4 shows the proteins that were different between the two groups. It is worth noting that in Figure 3 the stage IIb protein ratios ($\bar{X}1.675$; ±0.471) are greater than the stage IIa ratios ($\bar{X}1.431$; ±0.469) for the same proteins. Consequently, a paired *t*-test was performed comparing the two groups of values. The difference in the mean values between the two groups is greater than would be expected by chance; there is a statistically significant difference (*t* = −2.882;  *P* < .008). 

Tables 2 and 3 represent the up- (*n* = 34) and down- (*n* = 41) regulated proteins for the pooled saliva sample composed of individuals diagnosed with a Stage IIa IDC. The fold-increase of protein and *P*-values are also presented. As shown in Tables 2 and 3, 40 of the 75 proteins (53%) were significant at the *P *< .001 to *P *< .0001 levels.

Tables 4 and 5 are a list of the up- (*n* = 38) and down- (*n* = 45) regulated proteins observed in the Stage IIb cancer as compared to healthy controls. Of these 83 differentially expressed proteins, 54 (65%) were significant at the *P *< .001 to *P *< .0001 levels. There were 6 proteins that exhibited a 2.0 or greater fold increase in protein level in the Stage IIb cancer cohort as compared to the control subjects. 

## 4. Discussion

To the best of our knowledge this is the first attempt to determine salivary protein profile alterations related to lymphovascular invasion. As a consequence we have only a few references by which to compare our data. 

The proteins listed in Tables 2–4 are common to saliva and are listed in references concerning salivary proteomics of whole saliva and those constituents contributed by individual gland secretions [21, 31–34]. Likewise, many of the proteins are common to those identified in proteomic studies of cancer cell lines and serum or plasma from individuals diagnosed with IDC [21, 27, 35–39]. Additionally, there is a proteomic study by Pei et al. [40] that compared proteomic tissue protein profiles from paired normal tissue to malignant tissues with and without lymphovascular invasion [40]. Their results yielded 25 differentially expressed proteins among node positive and node negative, adenocarcinoma, colorectal cancer patients. From the list of 25 proteins that were differentially expressed when compared to a normal control by Pei et al., we matched 8 (32%) of them. These proteins were Apo-A1 protein, vimentin, cytokeratin-8, glutathione s-transferase, keratin 1, fructose-bisphosphate aldolase, alpha enolase, and transferring precursor [40]. Pei also reports Annexin II and IV while we found members Annexin I and III of the same family of proteins. Pei et al. identified four proteins which were differentially expressed. These were heat shock protein 27 (HSP-27), glutathione S-transferase (GST), Annexin II, and liver-fatty acid binding protein (L-FABP). The authors of this manuscript found 48 differentially expressed proteins (Figure 4) which included GST and a family member of the fatty acid binding proteins, epidermal-fatty acid binding protein. 

A second by Li et al. [41] used metastatic (lymph nodes) breast cancer cell lines that they developed in order to produce protein profile comparisons [41]. Comparative proteomic analysis using 2-DE and LC-IT-MS revealed that 102 protein gel spots were altered more than three-fold between the variant and its parental counterpart. Using SEQUEST with uninterpreted tandem mass raw data, they found eleven differentially expressed protein spots that were identified with high confidence. The proteins were identified as Cathepsin D precursor, peroxiredoxin 6 (PDX6), heat shock protein 27 (HSP27), HSP60, tropomyosin 1 (sent in the highly metastatic variant, whereas alpha B-crystalline (CRAB) was only detected in its parTPM1), TPM2, TPM3, TPM4, 14-3-3 protein epsilon, and tumor protein D54. The proteins were preental counterpart [41]. As shown in Tables 2–5, we identified a number of the same proteins. For example, the 14-3-3, tropomyosin and the peroxiredoxin family of proteins were found to be altered in the saliva of our late stage cancer profiles as well. Of particular interest is the fact that these proteins were not altered in the profiles of early stage cancer, that is, Stage 0 and Stage I performed by the authors of this manuscript [21, 42]. 

## 5. Conclusions

The authors have examined the salivary proteome that is altered in the presence of carcinoma of the breast with and without lymph node metastasis. We do not want to over emphasize the findings at this point, but we are encouraged to find that these protein profiles are found to be altered in the supernants from cancer tissues which provide additional support to our findings. 

The authors urge the exploration of saliva proteomics for in vivo systems modeling of carcinoma of tissues of ectodermal origin. Saliva can also be described as a media which provides “real-time” results [43]. The fluid is continually produced and excreted in an open-ended circuit, unlike blood which exists in a “closed-loop.” Blood, a circulating media, may contain proteins that are a day, a week, or a month old as well as proteins which have passed numerous times through many organ systems or have been excreted [43]. Saliva, with its continuous flow, is not subject to the aforementioned effects. Consequently, saliva and nipple aspirates may be a more useful than blood as the protein profiles of these fluids easier to assay than blood and are both altered in the presence of malignant diseases [16, 43]. 

Further study is required to determine their diagnostic utility. The authors plan to validate the protein biomarkers by western blot using commercially available antibodies. ELISA will also be used to assay a larger sample size and determine the sensitivity and specificity of the biomarkers. It is the hope of the investigators that this preliminary research will establish the foundation for a “point-of-care” test for clinical decision making in the treatment of carcinoma of the breast.

## 6. Disclosure Statement

The authors declare that they have no competing interests.

## Figures and Tables

**Table 1:** 

Summary of protein expression profiles			
Comparison	Up Regulated	Down Regulated	Total Markers
Stage IIa versus	34	41	75
healthy			
Stage IIb versus	38	45	83
healthy			
Totals	72	86	158

Stage IIa compared to stage IIb			

Common proteins	24	31	55
Differing proteins	24	24	48
Totals	48	55	83

**Table 2:** Upregulated salivary proteins for stage IIa (*n* = 34).

Accession	Gene ID	Name	Ratio	*P* value
P02763	A1AG1	Alpha-1 acid glycoprotein 1 precursor (AGP 1)	1.3229	.0127
P01023	A2MG	Alpha-2 macroglobulin precursor	1.1446	.0324
P07108	ACBP	AcylCoA binding protein	1.2264	.0254
P02768	ALBU	Serum albumin precursor	1.3677	.0000
P04745	AMYS	Salivary alpha amylase precursor	1.3564	.0000
P61769	B2MG	Beta-2 microglobulin precursor	1.1463	.0465
P04040	CATA	Catalase	1.0918	.0183
P01024	CO3	Complement C3 precursor	1.1698	.0194
P01040	CYTA	CystatinA (StefinA) (CystatinAS)	1.4403	.0005
P04080	CYTB	CystatinB (StefinB)	1.1440	.0108
P06733	ENOA	Alphaenolase	1.2626	.0000
Q01469	FABPE	Fatty acidbinding protein, epidermal (EFABP)	1.3126	.0022
Q5VTM1	fAM25	Protein FAM25	1.3647	.0165
P02765	FETUA	Alpha-2HS glycoprotein precursor	1.3101	.0129
P00738	HPT	Haptoglobin precursor	1.5562	.0000
P01781	HV320	Ig heavy chain VIII region GAL	1.3879	.0336
P01876	IGHA1	Ig alpha1 chain C region	1.4641	.0000
P01591	IGJ	Immunoglobulin J chain	1.2429	.0002
P35527	K1C9	Keratin, type I cytoskeletal 9	1.1629	.0279
P01834	KAC	Ig kappa chain C region	1.0953	.0016
P06870	KLK1	Kallikrein1 precursor	1.6991	.0000
P06309	KV205	Ig kappa chain VII region GM607 precursor	1.2300	.0131
P18135	KV312	Ig kappa chain VIII region HAH precursor	1.2637	.0077
P01842	LAC	Ig lambda chain C regions	1.0933	.0004
P31025	LCN1	Lipocalin1 precursor	1.3213	.0000
P80188	NGAL	Neutrophil gelatinase associated lipocalin precursor (Oncogene 24p3)	1.5325	.0000
P01833	PIGR	Hepatocellular carcinomaassociated protein TB6	1.3233	.0000
P26447	S10A4	Protein S100A4 (S100 calciumbinding protein A4)	1.1539	.0027
P31151	S10A7	Protein S100A7 (Psoriasin)	3.3466	.0000
P35321	SPR1A	Cornifin-A (19 kDa pancornulin)	1.4452	.0093
Q9UBC9	SPRR3	Cornifin beta (22 kDa pancornulin)	1.2787	.0000
P20061	TCO1	Transcobalamin1 precursor	1.1222	.0383
P62328	TYB4	Thymosin beta 4	2.1483	.0007
P62988	UBIQ	Ubiquitin	1.3427	.0424

**Table 3:** Downregulated salivary proteins for stage IIa (*n* = 41).

Accession	Gene ID	Name	Ratio	*P* value
P31947	1433S	14-3-3 protein sigma (Epithelial cell marker protein 1)	0.7921	.0043
P04075	ALDOA	Fructose-bisphosphate aldolase	0.7036	.0000
Q8N4F0	BPIL1	Lung and nasal epith, carcinoma-assoc. protein 2	0.7465	.0002
P23280	CAH6	Carbonic anhydrase 6 precursoranhydrase	0.7871	.0000
P54108	CRIS3	Cysteine-rich secretory protein 3 precursor (CRISP-3)	0.7846	.0003
P01034	CYTC	Cystatin-C precursor (Cystatin-3)	0.7108	.0000
P28325	CYTD	Cystatin-D precursor (Cystatin-5)	0.6047	.0000
P01037	CYTN	Cystatin-SN precursor	0.3200	.0000
P01036	CYTS	Cystatin-S precursor (Cystatin-4)	0.3385	.0000
P09228	CYTT	Cystatin-SA precursor (Cystatin-S5)	0.4153	.0000
Q9UGM3	DMBT1	Deleted in malignant brain tumors 1 protein precursor (Glycoprotein 340)	0.7910	.0050
P02671	FIBA	Fibrinogen alpha chain precursor [Contains: Fibrinopeptide A]	0.8964	.0104
P09211	GSTP1	Glutathione S-transferase P	0.8782	.0261
Q8IUE6	H2A2B	Histone H2A	0.5435	.0211
Q99880	H2B1L	Histone H2B type 1-L	0.5576	.0008
P62805	H4	Histone H4	0.4195	.0000
P15515	HIS1	Histatin-1 precursor	0.3152	.0340
P01857	IGHG1	Ig gamma-1 chain C region	0.9173	.0306
P13646	K1C13	Keratin, type I cytoskeletal 13 (Cytokeratin-13)	0.6395	.0011
P08779	K1C16	Keratin, type I cytoskeletal 16 (Cytokeratin-16)	0.6220	.0001
P61626	LYSC	Lysozyme C precursor	0.4121	.0000
P01871	MUC	Ig mu chain C region	0.6877	.0000
Q9HC84	MUC5B	Mucin-5B precursor	0.6404	.0000
P80303	NUCB2	Nucleobindin-2 precursor (Gastric cancer antigen Zg4)	0.6836	.0000
P22079	PERL	Lactoperoxidase precursor	0.8196	.0000
P05164	PERM	Myeloperoxidase precursor	0.6894	.0000
P12273	PIP	Prolactin-inducible protein precursor	0.6382	.0000
P23284	PPIB	Peptidyl-prolyl cis-trans isomerase B precursor	0.8769	.0326
P02812	PRB2	Basic salivary proline-rich protein 2	0.5422	.0000
Q06830	PRDX1	Peroxiredoxin-1	0.7296	.0020
P02810	PRPC	Salivary acidic proline-rich phosphoprotein 1/2 precursor	0.8848	.0000
P06703	S10A6	Protein S100-A6 (Growth factor-inducible protein 2A9)	0.8926	.0051
P05109	S10A8	Protein S100-A8 (S100 calcium-binding protein A8)	0.5888	.0000
P06702	S10A9	Protein S100-A9 (S100 calcium-binding protein A9)	0.6622	.0000
P31949	S10AB	Protein S100-A11 (S100 calcium-binding protein A11)	0.6526	.0017
Q96DR5	SPLC2	Lung and nasal epith. carcinoma-assoc. protein 2 precursor	0.3270	.0000
Q08188	TGM3	Protein-glutamine gamma-glutamyltransferase E precursor	0.7454	.0168
P29401	TKT	Transketolase	0.8236	.0351
P06753	TPM3	Tropomyosin alpha-3 chain	0.7809	.0016
P02788	TRFL	Lactotransferrin precursor	0.8118	.0000
P08670	VIME	Vimentin	0.7728	.0190

**Table 4:** Upregulated salivary proteins for stage IIb (*n* = 38).

Accession	Gene ID	Name	Ratio	*P* value
P02763	A1AG1	Alpha-1-acid glycoprotein 1 precursor (AGP 1)	1.8128	.0003
P01023	A2MG	Alpha-2-macroglobulin precursor (Alpha-2-M)	1.7279	.0000
P02768	ALBU	Serum albumin precursor	1.9149	.0000
P12429	ANXA3	Annexin A3 (Annexin III)	1.3144	.0058
P02647	APOA1	Apolipoprotein A-I precursor (Apo-AI)	1.2233	.0012
P00915	CAH1	Carbonic anhydrase 1	3.3424	.0356
P62158	CALM	Calmodulin (CaM)	1.5783	.0391
P04040	CATA	Catalase	1.4806	.0007
P01024	CO3	Complement C3 precursor	1.3391	.0000
P06733	ENOA	Alpha-enolase	1.4856	.0000
P02765	FETUA	Alpha-2-HS-glycoprotein precursor (Fetuin-A)	1.4004	.0007
P52566	GDIS	Rho GDP-dissociation inhibitor 2 (Rho GDI 2)	1.3866	.0046
P69905	HBA	Hemoglobin subunit alpha (Hemoglobin alpha chain)	1.8916	.0000
P68871	HBB	Hemoglobin subunit beta (Hemoglobin beta chain)	1.8326	.0000
P02790	HEMO	Hemopexin precursor (Beta-1B-glycoprotein)	2.2691	.0001
P00738	HPT	Haptoglobin precursor	2.3331	.0000
P01762	HV301	Ig heavy chain V-III region TRO	1.3350	.0262
P01781	HV320	Ig heavy chain V-III region GAL	1.6357	.0259
P01876	IGHA1	Ig alpha-1 chain C region	1.4384	.0000
P01877	IGHA2	Ig alpha-2 chain C region	1.1678	.0288
P01857	IGHG1	Ig gamma-1 chain C region	1.4955	.0000
P01859	IGHG2	Ig gamma-2 chain C region	1.5578	.0000
P01591	IGJ	Immunoglobulin J chain	1.3564	.0000
P01834	KAC	Ig kappa chain C region	1.2156	.0000
P06870	KLK1	Kallikrein-1 precursor	1.6222	.0000
P18135	KV312	Ig kappa chain V-III region HAH precursor	1.4020	.0009
P01842	LAC	Ig lambda chain C regions	1.3162	.0000
P31025	LCN1	Lipocalin-1 precursor	2.1725	.0000
P80188	NGAL	Neutrophil gelatinase-assoc. lipocalin precursor (Oncogene 24p3)	1.6181	.0000
P01833	PIGR	Hepatocellular carcinoma-associated protein TB6	1.2753	.0000
P31151	S10A7	Protein S100-A7 (S100 calcium-binding protein A7)	2.2683	.0000
P35321	SPR1A	Cornifin-A(SPR-IA)	1.6742	.0084
Q9UBC9	SPRR3	Cornifin beta (22 kDa pancornulin)	1.5506	.0000
P37837	TALDO	Transaldolase	1.3811	.0053
P20061	TCO1	Transcobalamin-1 precursor (Transcobalamin I)	1.2262	.0001
P02787	TRFE	Serotransferrin precursor (Transferrin)	1.5744	.0000
P62328	TYB4	Thymosin beta-4 (T beta 4)	3.3412	.0003
P62988	UBIQ	Ubiquitin	1.5925	.0055

**Table 5:** Downregulated salivary proteins for stage IIb (*n* = 45).

Accession	Gene ID	Protein Name	Ratio	*P* value
P31947	1433S	14-3-3 protein sigma (Stratifin) (Epithelial cell marker protein 1)	0.5486	.0000
P63104	1433Z	14-3-3 protein zeta/delta (Protein kinase C inhibitor protein 1)	0.5718	.0378
P04075	ALDOA	Fructose-bisphosphate aldolase A (Lung cancer antigen NY-LU-1)	0.7080	.0001
P19961	AMYC	Alpha-amylase 2B precursor	0.6840	.0020
P04745	AMYS	Salivary alpha-amylase precursor	0.9302	.0042
P04083	ANXA1	Annexin A1 (p35)	0.8105	.0080
P23280	CAH6	Carbonic anhydrase 6 precursor	0.4900	.0000
P10909	CLUS	Clusterin precursor (Complement-associated protein SP-40,40)	0.7704	.0252
P04080	CYTB	Cystatin-B (Stefin-B) (Liver thiol proteinase inhibitor)	0.8705	.0007
P01034	CYTC	Cystatin-C precursor (Cystatin-3)	0.5621	.0000
P28325	CYTD	Cystatin-D precursor (Cystatin-5)	0.4856	.0000
P01037	CYTN	Cystatin-SN precursor (Cystatin-1)	0.2323	.0000
P01036	CYTS	Cystatin-S precursor (Cystatin-4)	0.2437	.0000
P09228	CYTT	Cystatin-SA precursor (Cystatin-S5)	0.3033	.0000
Q02487	DSC2	Desmocollin-2 precursor (Desmosomal glycoprotein II and III)	0.7029	.0063
P06744	G6PI	Glucose-6-phosphate isomerase (SA-36)—(Human)	0.5118	.0013
P09211	GSTP1	Glutathione S-transferase P	0.7563	.0001
Q8IUE6	H2A2B	Histone H2A type 2-B	0.5699	.0058
Q99880	H2B1L	Histone H2B type 1-L (H2B.c)	0.6841	.0373
P62805	H4	Histone H4	0.3745	.0000
P15515	HIS1	Histatin-1 precursor (Histidine-rich protein 1)	0.2237	.0032
P30740	ILEU	Leukocyte elastase inhibitor (Serpin B1)	0.9203	.0418
P13646	K1C13	Keratin, type I cytoskeletal 13 (Cytokeratin-13)	0.4247	.0000
P08779	K1C16	Keratin, type I cytoskeletal 16 (Cytokeratin-16)	0.4900	.0000
P02538	K2C6A	Keratin, type II cytoskeletal 6A (Cytokeratin-6A)	0.6744	.0019
P61626	LYSC	Lysozyme C precursor	0.6646	.0000
P01871	MUC	Ig mu chain C region	0.7992	.0000
Q9HC84	MUC5B	Mucin-5B precursor (Mucin-5 subtype B, tracheobronchial)	0.9357	.0260
P80303	NUCB2	Nucleobindin-2 precursor (Gastric cancer antigen Zg4)	0.4934	.0000
P07237	PDIA1	Protein disulfide-isomerase precursor (p55)	0.8132	.0205
P22079	PERL	Lactoperoxidase precursor	0.6879	.0000
P05164	PERM	Myeloperoxidase precursor	0.7856	.0049
P12273	PIP	Prolactin-inducible protein precursor (gp17)	0.4659	.0000
P62937	PPIA	Peptidyl-prolyl cis-trans isomerase A	0.8031	.0091
P23284	PPIB	Peptidyl-prolyl cis-trans isomerase B precursor	0.7089	.0006
P02812	PRB2	Basic salivary proline-rich protein 2 (Salivary proline-rich protein)	0.4204	.0000
Q06830	PRDX1	Peroxiredoxin-1	0.6877	.0006
P02810	PRPC	Salivary acidic proline-rich phosphoprotein 1/2 precursor (PRP-1/PRP-2)	0.6861	.0000
P05109	S10A8	Protein S100-A8 (S100 calcium-binding protein A8) (Calgranulin-A)	0.8042	.0000
Q96DR5	SPLC2	Lung and nasal epith. carcinoma-assoc. protein 2 precursor	0.2322	.0000
P10599	THIO	Thioredoxin (Trx) (ATL-derived factor)	0.8003	.0003
P06753	TPM3	Tropomyosin alpha-3 chain (Tropomyosin-3)	0.8990	.0215
P02788	TRFL	Lactotransferrin precursor	0.7072	.0000
P08670	VIME	Vimentin	0.7717	.0277
P25311	ZA2G	Zinc-alpha-2-glycoprotein precursor (Zn-alpha-2-glycoprotein)	0.8543	.0000
